# Protein Palmitoylation Modification During Viral Infection and Detection Methods of Palmitoylated Proteins

**DOI:** 10.3389/fcimb.2022.821596

**Published:** 2022-01-27

**Authors:** Xiaoling Li, Lingyi Shen, Zhao Xu, Wei Liu, Aihua Li, Jun Xu

**Affiliations:** ^1^ College of Life Sciences, Henan Agricultural University, Zhengzhou, China; ^2^ Clinical Lab, Henan Provincial Chest Hospital, Zhengzhou, China

**Keywords:** post-translational modification, S-palmitoylation, viral infection, virus-host interaction, detection methods

## Abstract

Protein palmitoylation—a lipid modification in which one or more cysteine thiols on a substrate protein are modified to form a thioester with a palmitoyl group—is a significant post-translational biological process. This process regulates the trafficking, subcellular localization, and stability of different proteins in cells. Since palmitoylation participates in various biological processes, it is related to the occurrence and development of multiple diseases. It has been well evidenced that the proteins whose functions are palmitoylation-dependent or directly involved in key proteins’ palmitoylation/depalmitoylation cycle may be a potential source of novel therapeutic drugs for the related diseases. Many researchers have reported palmitoylation of proteins, which are crucial for host-virus interactions during viral infection. Quite a few explorations have focused on figuring out whether targeting the acylation of viral or host proteins might be a strategy to combat viral diseases. All these remarkable achievements in protein palmitoylation have been made to technological advances. This paper gives an overview of protein palmitoylation modification during viral infection and the methods for palmitoylated protein detection. Future challenges and potential developments are proposed.

## 1 Introduction

Protein lipidation is an important post-translational modification in which lipid moieties are covalently attached to proteins. This process increases the hydrophobicity of proteins, thereby resulting in changes in protein conformation, stability, membrane association, localization, and binding properties. Palmitoylation refers to the covalent modification of proteins with palmitoyl groups. S-palmitoylation of proteins, which is one of the most common forms of palmitoylation, occurs when saturated C16 fatty acids covalently attach to the side chains of cysteine residues through unstable thioester-bonds (Fukata and Fukata, 2010; Blanc et al., 2013; Hentschel et al., 2016; Resh, 2016; Zaballa and van der Goot, 2018). Studies have shown the existence of more than 40 different molecular types of free fatty acids, including a variety of long-chain/short-chain, saturated/unsaturated acids, that can participate in the esterification of proteins (Quehenberger et al., 2010). Palmitoylation of proteins is a reversible dynamic process, and depalmitoylation occurs when, under certain conditions, the thioester bond is hydrolyzed, and the palmitate is separated from the cysteine residue. This kind of reversible modification is present in all eukaryotes, including mammals, plants (Hemsley, 2017), and parasites (Blanc et al., 2013; Brown et al., 2017; Corvi and Turowski, 2019); it regulates trafficking (Linder and Deschenes, 2007; Daniotti et al., 2017; Ernst et al., 2018; Tortosa and Hoogenraad, 2018; Sun et al., 2020), localization (Gamage et al., 2017; Sada et al., 2019; Nakamura et al., 2020), stability (Gok et al., 2020; Wang et al., 2020), interaction (Zaballa and van der Goot, 2018), and signal transduction of proteins (Zheng et al., 2012; Globa and Bamji, 2017; Zingler et al., 2019).

Furthermore, palmitoylation modification is associated with various kinds of diseases, such as neurological diseases (Alzheimer’s disease, Huntington’s disease, and schizophrenia) (Sanders and Hayden, 2015; Cho and Park, 2016), infectious diseases, and even cancer (Blanc, Blaskovic et al., 2013; Hornemann, 2015; Resh, 2017). In recent years, considerable reports have shown that the glycoproteins of almost all of the enveloped viruses have at least one S-acylated site (Gadalla and Veit, 2020). Also, the role played by palmitoylated viral proteins in the infection process has been discussed from different angles (Veit, 2012; Veit et al., 2013; Sobocinska et al., 2017). Therefore, it is important to develop reliable and practical detection methods to analyze the palmitoylation modification of proteins. Researchers have utilized the latest technologies and have made considerable progress in exploring the pathogenesis of diseases related to palmitoylation (Brigidi and Bamji, 2013; Gu and Robinson, 2016; Gao and Hannoush, 2018; Peng and Hang, 2019). However, in the last decade, only a few research teams have systematically reviewed the detection methods of palmitoylation (Lanyon-Hogg et al., 2017; Gao and Hannoush, 2018; Zaballa and van der Goot, 2018; Lu and Fang, 2020). This paper reviewed the palmitoylation modification and the different analytical methods to detect palmitoylated proteins. The palmitoylation of proteins related to viral infection was emphasized, and future research prospects were reviewed.

## 2 Protein Palmitoylation/Depalmitoylation Cycle

### 2.1 S-Palmitoylation

S-palmitoylation is a post-translational modification of proteins resulting from the covalent attachment of the palmitoyl chain to a cysteine residue(s) of proteins through a reversible thioester bond. This unique reversible process can act as a molecular switch, just like phosphorylation or ubiquitination (Blaskovic et al., 2013). Due to the obvious heterogeneity with other fatty acids linked to cysteine residues, the fatty acylation process is generally known as “thioacylation” or “S-acylation” rather than palmitoylation (Linder and Deschenes, 2007). Although “S-palmitoylation” implies that cysteine residues are only modified by 16:0 fatty acids, in the cell, they can also be modified by long-chain fatty acids with different degrees of unsaturation (Thinon et al., 2016; Greaves et al., 2017). S-palmitoylation of some proteins (e.g., cytosolic proteins) helps them acquire hydrophobic anchors, resulting in an improved association with membrane, trafficking, and localization. In many cases, palmitoylation of key cellular transmembrane proteins also occurs, including adhesion molecules, tetraspanins, G-protein-coupled receptors, receptor ligands, and viral glycoproteins (Blaskovic et al., 2013). In theory, palmitates should be added to the N-terminal of cysteine residues—known as N-palmitoylation—to form a more stable and irreversible structure. However, N-palmitoylation is rare in natural viral proteins, and its biological function has not been explored so far. Therefore, unless specified, palmitoylation of proteins usually refers to S-palmitoylation at the cysteine site.

Since the identification of the first protein to have undergone S-palmitoylation, nearly a thousand eukaryotic proteins have been identified as the substrates of palmitoylation (Blanc et al., 2015; Blanc et al., 2019). Some experts in this field have reviewed the roles of S-palmitoylation in synaptic plasticity, membrane trafficking, and physiological processes, such as metabolism, cell death, cell polarity and migration, cancer, and innate immunity (Chamberlain and Shipston, 2015; Daniotti et al., 2017; Zareba-Koziol et al., 2018); therefore, this part will not be discussed here.

### 2.2 Palmitoylation/Depalmitoylation: A Dynamic Reversible Cycle

**Figure 1:**
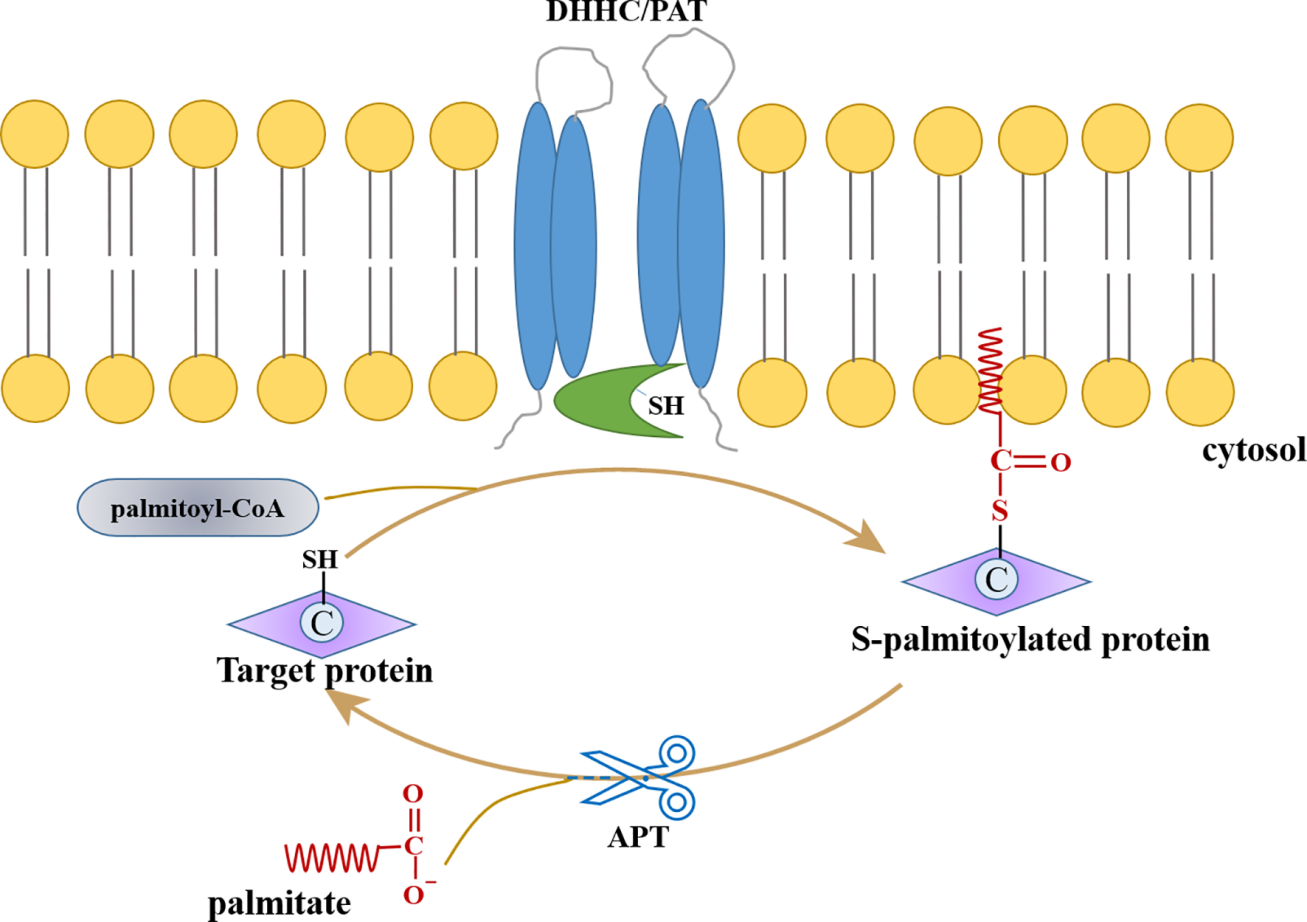
Protein palmitoylation/depalmitoylation cycle.

S-palmitoylation is a reversible and dynamic modification; palmitoyl acyltransferases (PATs) catalyze the addition of palmitic acid, while the removal of palmitic acid is mediated by acyl protein thioesterases (APTs) (**Figure 1**). Since PATs contain Zn^2+^ binding domains and conserved “Asp-His-His-Cys” (DHHC) motifs, they are considered to be part of the zDHHC group of enzymes (Mitchell et al., 2006; Gottlieb and Linder, 2017). The first discovered palmitoyl transferases are Erf2p and Erf4p of *Saccharomyces cerevisiae* and are identified as Ras protein acyltransferases (Ras PAT). Erf2p, containing conserved DHHC-cysteine-rich domain, forms a complex with Erf4p to function as the effectors of Ras protein in the endoplasmic reticulum (ER) membrane (Lobo et al., 2002; Roth et al., 2002). Since then, twenty-three human DHHC proteins have been identified or predicted to form a protein superfamily.

The zDHHC enzymes, usually present in Golgi membrane or ER, catalyzes the palmitoylation process of substrate proteins; this is a two-step process, where palmitate is transferred onto the thiol group of cysteine from cytosolic palmitoyl-CoA by PAT. First, the Asp and His act as a proton shuttle, thereby converting the Cys residue of the zDHHC enzyme into a thiolate nucleophile. In the second step, the carbonyl carbon of the palmityl-CoA thioester is attacked by Cys thiolate, resulting in instantaneous auto-acylation of cysteine residues in its DHHC motif. This facilitates the PAT-mediated transfer of the palmitoyl residue to the substrate protein (Stix et al., 2020) (**Figure 1**). The mutational analysis has revealed that the first histidine of the DHHC motif is especially important during the second step (Mitchell et al., 2010; Jennings and Linder, 2012; Tsai et al., 2014; Gonzalez Montoro et al., 2015). Recent research by Rana et al. revealed the crystal structures of two DHHC palmitoyl transferases leading to an understanding that the active site resides at the membrane-cytosol interface. Based on this, it was concluded that the thioester-exchange reaction is catalyzed by DHHC enzymes and that the membrane-proximal cysteines are potential sites for palmitoylation (Rana et al., 2018; Rana et al., 2019; Stix et al., 2020).

Compared to palmitoylation, i.e., its reverse reaction, depalmitoylation, is poorly understood; and, only seven APTs have been identified to catalyze depalmitoylation. APT (commonly referred to as palmitoyl protein thioesterase or PPT) catalyzes the hydrolysis of thioesters to dissolve and replace the substrate protein on the membrane. Structural studies show that two depalmitoylases (APT1 and APT2), which were identified earlier (Duncan and Gilman, 1998; Toyoda et al., 1999), contain convergent acyl binding channels, indicating that factors other than acyl chain recognition also mediate the selection of substrate (Devedjiev et al., 2000). Recombinant PPT1 enzyme from baculovirus expression system acts as a powerful depalmitoylase for H-Ras protein (Camp and Hofmann, 1993). The mammalian α/β hydrolase-domain containing protein (ABHD) recently became the novel candidate for regulating lipid metabolism. ABHD17A and its two isoforms, ABHD17B and ABHD17C, were identified as depalmitoylases targeting Ras-family GTPases and synaptic proteins in neurons (Lin and Conibear, 2015). Moreover, another depalmitoylase, ABHD10, is known to act on S-palmitoylated peroxiredoxin-5 protein and regulates redox homeostasis in mitochondria (Cao et al., 2019).

The reversibility of protein palmitoylation modification suggests a subtle mechanism, which avoids imbalance. To date, quite a few reports have demonstrated the relationship between the defects of palmitoylation/depalmitoylation process and human diseases, including Huntington’s disease (Sanders and Hayden, 2015), schizophrenia (Ota et al., 2013), Alzheimer’s disease (Mizumaru et al., 2009; Vetrivel et al., 2009), Goltz syndrome (Biechele et al., 2011; Doubravska et al., 2011), and other diseases (Yeste-Velasco et al., 2015; Ko and Dixon, 2018) related to PATs. However, it is still unclear how the cycle works in detail, especially, which PAT participates or maintains this dynamic equilibrium of the target proteins involved in the above diseases.

## 3 Protein Palmitoylation Modification During Viral Infection

### 3.1 Palmitoylation of Viral Proteins

Many cell-and-animal-based studies have reported the multiple roles of protein modifications in viral infectious diseases. Palmitoylation of viral proteins, which are involved in virus assembly or pathogenesis, has been found in most of the eukaryotic permissive cell types, including yeast, insect, plant, and vertebrate cells (Veit, 2012; Blanc et al., 2013; Lin et al., 2017). In **Table 1**, we have summarized the viral proteins, especially structural proteins of virus envelope that undergo palmitoylation during viral infections leading to serious diseases in humans or animals. Also, we have provided select examples illustrating key findings and the important roles of palmitoylated viral proteins during viral infections.

**Table 1:** Summary of some palmitoylated viral proteins reported..

Virus	genome	Family	Protein Name	Type of membrane protein	Number of Cysteine sites palmitoylated	References (Name/year)
Flu A	−ssRNA	Orthomyxovirus	M2	Type III membrane proteins	1	(Grantham et al., 2009; Thaa et al., 2011; Thaa et al., 2012; Veit and Siche, 2015; Ekanayake et al., 2016; Manzoor et al., 2017; Su et al., 2018)
			HA	Type I glycoproteins	3	(Melkonian et al., 1999; Kordyukova et al., 2008; Engel et al., 2010; McBride and Machamer, 2010; Kordyukova et al., 2011; Brett et al., 2014; Chlanda et al., 2017; Gadalla et al., 2020)
Flu B	−ssRNA	Orthomyxovirus	HA	Type I glycoproteins	2	(Ujike et al., 2004; Ujike et al., 2005; Gadalla et al., 2020)
			NB	Type III membrane proteins	1	(Hatta and Kawaoka, 2003; Demers et al., 2014)
Flu C	−ssRNA	Orthomyxovirus	CM2	Type III membrane proteins	1	(Muraki et al., 2011; Muraki et al., 2013; Goto et al., 2017)
Measles virus	−ssRNA	Paramyxovirus	F	Type I glycoproteins	5	(Caballero et al., 1998; Branigan et al., 2006)
hRSV	−ssRNA	Paramyxovirus	F	Type I glycoproteins	1	(Caballero et al., 1998; Branigan et al., 2006; Ohol et al., 2015)
Sindbis virus	+ssRNA	Togavirus	E1	Type I glycoproteins	1	(Ryan et al., 1998)
			E2	Type I glycoproteins	5	(Ivanova and Schlesinger, 1993; Ryan et al., 1998; Wilkinson et al., 2000; Ramsey et al., 2019)
			6K	Membrane proteins with a hairpin topology	5	(Gaedigk-Nitschko and Schlesinger, 1990; Ramsey et al., 2017)
SFV (Semliki Forest virus)	+ssRNA	Togavirus	E2	Type I glycoproteins	4	(Kordyukova et al., 2010)
			6K	Membrane proteins with a hairpin topology	5	(Kordyukova et al., 2010)
VSV	−ssRNA	Rhabdovirus	G	Type I glycoproteins	1	(Schmidt and Schlesinger, 1979; Whitt and Rose, 1991; Kordyukova et al., 2010)
Rabies virus	−ssRNA	Rhabdovirus	G	Type I glycoproteins	1	(Gaudin et al., 1991)
Ebola virus	−ssRNA	Filovirus	Gp	Type I glycoproteins	2	(Ito et al., 2001)
Marburg virus	−ssRNA	Filovirus	Gp	Type I glycoproteins	2	(Funke et al., 1995; Ito et al., 2001)
MHV	+ssRNA	Coronavirus	S	Type I glycoproteins	9	(Thorp et al., 2006; Gelhaus et al., 2014)
			E	Type III membrane proteins	3	(Boscarino et al., 2008; Lopez et al., 2008)
SARS-CoV-2	+ssRNA	Coronavirus	S	Type I glycoproteins	10	(Nguyen et al., 2020; Mesquita et al., 2021; Puthenveetil et al., 2021; Wu et al., 2021; Zeng et al., 2021)
			E	Type III membrane proteins	3	(Abdulrahman et al., 2021)
SARS-CoV	+ssRNA	Coronavirus	S	Type I glycoproteins	9	(Petit et al., 2007; Akerstrom et al., 2009; Shulla and Gallagher, 2009; McBride and Machamer, 2010; Gelhaus et al., 2014)
			E	Type III membrane proteins	3	(Liao et al., 2006; Tseng et al., 2014)
HIV (HXB2D)	+ssRNA	Retrovirus	Gp	Type I glycoproteins	2	(Yang et al., 1995; Rousso et al., 2000; Bhattacharya et al., 2004; Yang et al., 2010)
HCMV	dsDNA	Herpesviruses	Gn	Type I glycoproteins	2	(Mach et al., 2007)
			Gb	Type I glycoproteins	1	(Patrone et al., 2016)

dsDNA, double-stranded DNA; +ssRNA, positive-sense, single-stranded RNA; −ssRNA, negative-sense, single-stranded RNA.

Since the identification of palmitoylated glycoproteins of Sindbis virus and Vesicular stomatitis virus in 1979 (Schmidt et al., 1979; Schmidt and Schlesinger, 1979), many other palmitoylated proteins of viruses have been reported, such as hemagglutinin (HA) and proton channel M2 of influenza virus, glycoproteins of filoviruses and retrovirus (including HIV), fusion protein (F) of measles virus, the S-protein of coronavirus (CoV) (Veit, 2012; Kordyukova et al., 2019). It is noteworthy that the palmitoylation sites of SARS-CoV-2 spike protein have recently been identified and proved to be essential for SARS-CoV-2 fusion with the host cell (Mesquita et al., 2021; Puthenveetil et al., 2021). Palmitoylation modification of proteins may facilitate trafficking of glycoproteins on viral membranes, thereby promoting assembly and budding of progeny virions on infected epithelial cells (Veit et al., 2013; Demers et al., 2014). Moreover, the palmitoylation of non-structural viral proteins, such as Chikungunya virus (CHIKV) nsP1 and hepatitis C virus (HCV) NS2, are crucial to the successful infection (Utt et al., 2019; Wu et al., 2019; Zhang et al., 2019; Bakhache et al., 2020). In mammalian cells, CHIKV replication complex anchors on the plasma membrane. Palmitoylation of the cysteine residue at the C-terminus of CHIKV nsP1 enhances its interaction with the lipid bilayer, which is critical for targeting cholesterol-rich lipid rafts and viral genome replication (Utt et al., 2019; Zhang et al., 2019). The subcellular localization of HCV NS2 is regulated through palmitoylation, which also promotes the replication of HCV RNA. Therefore, palmitoylation of HCV NS2 could be utilized to inhibit HCV RNA replication and viral assembly, providing a novel alternative strategy effective against HCV infection (Wu et al., 2019).

Besides the above-mentioned mammalian viruses, in the viruses infecting aquatic animals and plants, palmitoylation of proteins plays an important role in viral replication, protein localization, and other related functions. A recent study reported that exogenous palmitic acid promotes red-spotted grouper nervous necrosis virus (RGNNV) infection, and interfering with the palmitoylation and phospholipid synthesis significantly inhibits RGNNV replication (Huang et al., 2020). In plant cells, the S-palmitoylation of Mungbean yellow mosaic virus (MYMV) AC4 allows its accumulation on the plasma membrane, thereby counteracting the host defense mechanism by suppressing the post-transcriptional gene silencing (PTGS) (Carluccio et al., 2018).

In fact, another important function of palmitoylation is to target modified proteins to lipid rafts (Diaz-Rohrer et al., 2014; Stepanek et al., 2014). These rafts provide the membrane platform for the entry, assembly, and budding of different viruses. It is speculated that the specific inhibition of acyltransferases that dramatically inhibits viral replication will not affect the palmitoylation of intracellular proteins because the transportation of fatty acids could be mediated by other members of the zDHHC family (Veit and Siche, 2015; Gadalla and Veit, 2020). Therefore, some researchers proposed that inhibition of certain protein palmitoylation may be a promising strategy for treating related diseases (Chavda et al., 2014; Lanyon-Hogg et al., 2017; Lin et al., 2017; Chen et al., 2018). The zDHHC protein family and APTs could be utilized as key modifiers of protein palmitoylation cycles, making them potential drug targets, which means ZDHHCs targeting viral proteins need to be identified (Gadalla and Veit, 2020). However, although palmitoylation of viral proteins had already been discovered 43 years ago (Schmidt and Schlesinger, 1979), the identification of PATs responsible for S-acylation of viral proteins remained a major gap till several zDHHCs catalyzing the S-acylation of proteins of influenza viruses and SARS-CoV-2 were found. Using CRISPR/Cas9 knockout and siRNA screening, DHHC2, 8, 15, and 20 were found to be involved in S-acylation of HA and M2 of IAV and human influenza viruses (Gadalla et al., 2020). DHHC20, -8, and -9 were verified to play certain roles in SARS-CoV-2 spike protein(S) and enveloped protein (Abdulrahman et al., 2021; Mesquita et al., 2021; Puthenveetil et al., 2021). However, conflicting results regarding the palmitoylation sites and palmitoylation enzymes of SARS-CoV-2 spike protein were obtained by different researchers (Mesquita et al., 2021; Puthenveetil et al., 2021; Zeng et al., 2021; Li et al., 2022). Li et al. found the cysteines of C-terminal and N‐terminal C15 of the spike protein were palmitoylated (Li et al., 2022), while Wu et al. reported that N-terminal C15 mutant did not affect the S-acylation of the spike (Wu, Zhang et al., 2021). Using computational, lipidomic, and biochemical approaches, Mesquita et al. deciphered the roles of zDHHC20 and zDHHC9 in the palmitoylation of the C terminal cysteines of spike protein in *in vivo* and *in vitro* conditions (Mesquita et al., 2021). The reasons for the conflicting results may be the use of different viruses and cell models or different methods. In addition no specific molecule drug for inhibiting a certain zDHHC substrate interaction has been developed so far (Abdulrahman et al., 2021). Looking forward, we believe that the discovery and identification of enzymes and their specific inhibitors acting on the palmitoylation cycle of viral proteins would be a new challenge and almost certainly would become one of the most interesting topics to investigate. Further biochemical and structural analysis of different zDHHCs and a series of different viral and cellular substrates are needed to understand the mechanism of acylation reactions (Gadalla and Veit, 2020).

### 3.2 Palmitoylation of Host Cell Proteins

Besides the expression of immune-related genes, viral infection can trigger a series of immune responses in host cells, including the modification of related proteins. However, so far, few such proteins have been reported. Furthermore, the palmitoylated host proteins that have been reported during viral infection are associated with interferon (IFN) pathways, e.g., stimulator of interferon genes (STING) and interferon-induced transmembrane (IFITM).

STING, a central signal component of DNA sensing pathways in cells, is a class of small molecular proteins located in the endoplasmic reticulum (Ishikawa et al., 2009; Sun et al., 2013). It is the optimal inducer of type I IFN in response to invading viruses or bacteria (Ishikawa and Barber, 2008; Hansen et al., 2014; Christensen et al., 2016; Holm et al., 2016). Excessive activation of the STING signaling pathway gives rise to abnormal responses of the innate immune system, leading to a series of autoinflammatory diseases (Hansen et al., 2019). Researchers have found that STING activation depends on its palmitoylation in the Golgi complex (Ishikawa and Barber, 2008). A non-specific inhibitor for protein palmitoylation, 2-bromopalmitate (2-BP), can inhibit palmitoylation of STING and eliminate type I IFN response (Mukai et al., 2016). Another important finding is the identification of a class of highly effective and selective antagonists blocking STING palmitoylation by covalently binding to the predicted transmembrane Cys91 (Mukai et al., 2016). Experimental evidence showed the antagonists, and their derivatives can down-regulate STING-mediated inflammatory cytokine expression (Haag et al., 2018). Together, these data imply the potential of STING-palmitoylation-targeted therapy in the treatment of autoinflammatory diseases.

Another broad-spectrum antiviral protein family known as IFITMs has recently emerged as promising palmitoylation targets (Brass et al., 2009; Yount et al., 2010; Yount et al., 2012; Diamond and Farzan, 2013; McMichael et al., 2017). The cysteine palmitoylation of IFITMs plays an important role in their antiviral activities because this modification may help in the regulation of IFITMs localization (Hach et al., 2013). Besides, localization in the vesicle membrane allows IFITM to directly fuse with the specific virus particles. This fusion complex is then trafficked to the lysosome, which relies on the palmitoylation of its three conserved cysteines at 70,71, and 105 sites (Spence et al., 2019). Furthermore, preventing the fusion of the viral membrane with the cells or the fusion among the infected cells could be the main antiviral mechanism of IFITM (Buchrieser et al., 2019; Zani et al., 2019). A very interesting study showed that placental damage and abortion caused by the virus-induced upregulation of type I IFN during pregnancy is associated with the palmitoylation of IFITM3. This can be caused by the mutation in three cysteine residues of IFITM3, which suppresses the inhibition of cell fusion (Buchrieser et al., 2019). In one of our studies, we confirmed that resistance to the Japanese encephalitis virus (JEV) infection requires the S-palmitoylation modification of IFITM in swine (Xu et al., 2020). Therefore, the discovery of activators or inhibitors targeting the palmitoylation/depalmitoylation cycle of IFITM would be a promising subject for antiviral drugs or immunomodifiers of the diseases caused by viral infections or immune disorders.

## 4 Detection Methods for Palmitoylation Modifications

The researchers have developed different approaches to detect S-palmitoylation, which has effectively contributed to the identification and functional research. This section summarizes the principles and brief procedures of these methods and discusses their major limitations. According to the different targets of analysis in the S-palmitoyl group, the identification methods can be divided roughly into two categories, the palmitate-targeted and the cysteine-targeted (also called lipid-and cysteine-centric). All these techniques are label-based, except the technique of gas chromatography-mass spectrometry (GC-MS) and MALDI-TOF MS, which is a relatively direct way. When palmitic acid is the target of analysis, it is usually labeled with an analog of palmitic acid or a probe, and then the labeled proteins are enriched by affinity purification or observed with optical instruments. However, such methods cannot be applied to body fluids or tissue samples of *in vivo* experiments because it requires the metabolic activity of the radioactive label or chemically modified palmitic acid analogs. Besides, when palmitic acid analog is labeled on the target protein, other shorter, longer, or unsaturated fatty acid modifications cannot be analyzed except for palmitoylated modifications. In the cysteine-centric method, biotin or molecular mass labels are utilized to detect palmitoylation proteins by affinity enrichment purification or western blotting. Because this method does not require metabolic labeling in live cells, it can be used to analyze not only samples of cells but also tissues and body fluids. The general operating procedures of the commonly used strategies mentioned above are described separately.

### 4.1 Mass Spectrometry

#### 4.1.1 Gas Chromatography–Coupled Mass Spectrometry

GC-MS is a relatively direct and reproducible method and has been widely used for analyzing hundreds of metabolites in biological fluids or tissue samples (Zeki et al., 2020). The method mainly comprises the following steps: 1) preparation of purified protein samples; 2) washing the samples to remove non-covalently coupled lipids; 3) hydrogenation with hydroxylamine for cleavage of thioester bonds and simultaneous ethyl esterification with anhydrous formic acid and ethanol; 4) liquid phase extraction to separate free fatty acids; and 5) the detection of the exact lipid fraction with GC-MS (Sorek and Yalovsky, 2010; Sorek et al., 2013). Based on GC-MS analysis, the purified recombinant Rho-related GTPase ROP6 (Thomas et al., 1992) and calcium sensor protein CBL1 (Uchida and Stadtman, 1992) of *Arabidopsis* have been identified as C-16 palmitic acid and C-18 stearic acid-modified proteins, respectively (Batistic et al., 2008; Sorek et al., 2011; Sorek et al., 2013). Given the range of protein concentration and volume of samples required for GC-MS analysis, S-palmitoylation can be detected for a purified protein sample with a concentration as low as 1 µg (Li et al., 2011). However, to a certain extent, the application of GC-MS in the identification of palmitoylated proteins is limited because of its need for purified protein samples. Since most of the palmitoylated proteins are membrane-localized and have strong hydrophobicity, their purification is a difficult process.

#### 4.1.2 MALDI-TOF Mass Spectrometry

Matrix-assisted laser desorption ionization-time of flight (MALDI-TOF) mass spectrometry (MS) is the only method that allows detecting directly the exact cysteine binding of fatty acid and the type of fatty acid attached to the S-acylated hydrophobic molecules (Webster and Oxley, 2012). In addition, this approach could be applied to the proteins that are difficult purify from protein mixtures. Besides the identification of palmitoylation, the MALDI-TOF MS-based approach revealed lipidation modifications of several cellular proteins and the heterogeneous fatty acylation of neuromodulin GAP-43 and GNAI (Liang et al., 2002) (Nuskova et al., 2021).

Further, by this method, the differential S-acylation of many viral proteins was confirmed (Serebryakova et al., 2006; Kordyukova et al., 2008; Kordyukova et al., 2010; Mineev et al., 2013; Vitale et al., 2013; Brett et al., 2014). Therefore, MALDI-TOF MS is an approach that uniquely detects hydrophobic viral lipopeptides, which assist in finding therapeutic targets to prevent viruses.

In contrast to MALDI-MS, which is perfect to identify the exact type of fatty acid attached to the exact S-acylation site, the LC-MS/MS based methods are adopted mainly in combination with other approaches, for example, ssABE (Collins et al., 2017; Woodley and Collins, 2019) and PalmPISC (Yang et al., 2010; Dowal et al., 2011) are all combined with acyl-biotin exchange (ABE) method, which will be mentioned below.

### 4.2 Radioactive Palmitic Acid Metabolic Labeling

Radioactive labeling has been a relatively straightforward approach because the palmitoyl labeled with radioisotope ^3^H or ^14^C could be developed by autoradiography. Following metabolic labeling of the samples, the protein can be easily immunoprecipitated and analyzed using SDS-PAGE; the radiolabeled target protein can be visualized by autoradiography (Peseckis et al., 1993; Swarthout et al., 2005; Zou et al., 2011; Tsai et al., 2014). The main steps of this approach include: 1) incubating the cultured cells in the serum-free medium; 2) labeling cells with radiolabeled ^3^H-palmitic acid; 3) washing cells to remove unbound ^3^H-palmitic acid; 4) immunoprecipitation and analysis of the labeled proteins by SDS-PAGE, and 5) visualization of the ^3^H emission signal by X-ray film exposure. This method was utilized to identify the palmitoylation of influenza A glycoprotein hemagglutinin (HA) and hepatitis E virus (HEV) ORF3 protein (Gouttenoire et al., 2018; Gadalla et al., 2020). However, since this method is less sensitive, tedious, time-consuming, and hazardous (because of radioactive material), it has not been used very often in recent years (Lu and Fang, 2020).

### 4.3 Click Chemistry

Click chemistry provides an alternative way to analyze S-palmitoylated proteins. By means of Huisgen’s copper (I)-catalyzed azide-alkyne cycloaddition reaction, target proteins are labeled with terminal azide or alkynyl groups, purified by affinity chromatography, and identified with mass spectrometry or gel-based visualization (Speers and Cravatt, 2004; Sobocinska et al., 2017). The commercially available alkyne fatty acid analog, 17-octadecynoic acid (17-ODYA), is commonly used as a non-radioactive derivative of palmitic acid. This is because it can enter into palmitoylated endogenous sites by metabolic pathways of the cellular palmitoylation mechanism. In addition, based on the biological orthogonal method (this allows multiple methods of labeling to be used in the same biosystems), dual-click chemistry pulse-chase scheme has been developed. For example, both 17-ODYA and methionine surrogate L-azidohomoalanine (L–AHA) are used for detecting dynamic palmitoylation modification and monitoring the turnover rate of proteins (Lin and Conibear, 2015). Besides, the click chemistry-based method can be used to study the subcellular localization of palmitoylated proteins through *in situ* probe combination techniques (Gao and Hannoush, 2014; Jiang et al., 2018; Stypulkowski et al., 2018). Also, if combined with mass spectrometry-based proteomics, this kind of non-radioactive labeling of alkynyl fatty acids is far more sensitive and safety, making it easier to analyze the palmitoylated protein globally and dynamically (Charron et al., 2009; Martin and Cravatt, 2009; Yount et al., 2010; Martin et al., 2011). However, under *in situ* environment, analogs of palmitic acid may hinder the normal metabolism and other processes because of the complexity of eukaryotic cell metabolism; for example, N-, and O-palmitoylation and N-myristoylation sites of proteins could reduce the efficiency and accuracy of detection methods (Jones et al., 2012; Gao and Hannoush, 2014; Wright et al., 2014).

### 4.4 Acyl-Biotin Exchange (ABE) and Acyl-Resin Assisted Capture (acyl-RAC)

These two methods are cysteine-centric and involve non-radioactive labeling, and to some extent, simplify the identification and quantification of palmitoylated proteins. These methods include several key steps: completely blocking the free sulfhydryl group, using hydroxylamine to hydrolyze all thioester bonds, and capturing the newly released free sulfhydryl group with a pyridyl-disulfide bond biotin conjugate (Forrester et al., 2011; Guo et al., 2014) to form biotin linked by disulfide bonds (ABE), or directly capturing them by pyridyl disulfide bond resin (Acyl-RAC). For the detection of S-palmitoylation, the peptides labeled with biotin are enriched with streptavidin resin, and then subjected to mass spectrometry.

Since ABE and acyl-RAC are easier, convenient, and timesaving, by far, hundreds of S-palmitoylated proteins have been identified, and their S-palmitoylated sites have been annotated (Linder et al., 1993; Mumby and Muntz, 1995; Forrester et al., 2011; Zhou et al., 2019). However, there are certain disadvantages of ABE, including the high background caused by captured non-S-palmitoylated proteins and the fact that the type of lipid attached to the protein cannot be accurately identified and needs further analysis. Furthermore, these indirect detection methods cannot accurately quantify the palmitoylation sites of endogenous proteins. By adding 2,2′-dithiodipyridine, Zhou et al. developed a low-background ABE (LB-ABE) method based on the blocking of residual free cysteine residues before the biotin-HPDP reaction and identified thousands of candidate S-palmitoylated proteins (Zhou et al., 2019).

### 4.5 Acyl-PEGyl Exchange Gel-Shift (APEGS) Method

**Figure 2:**
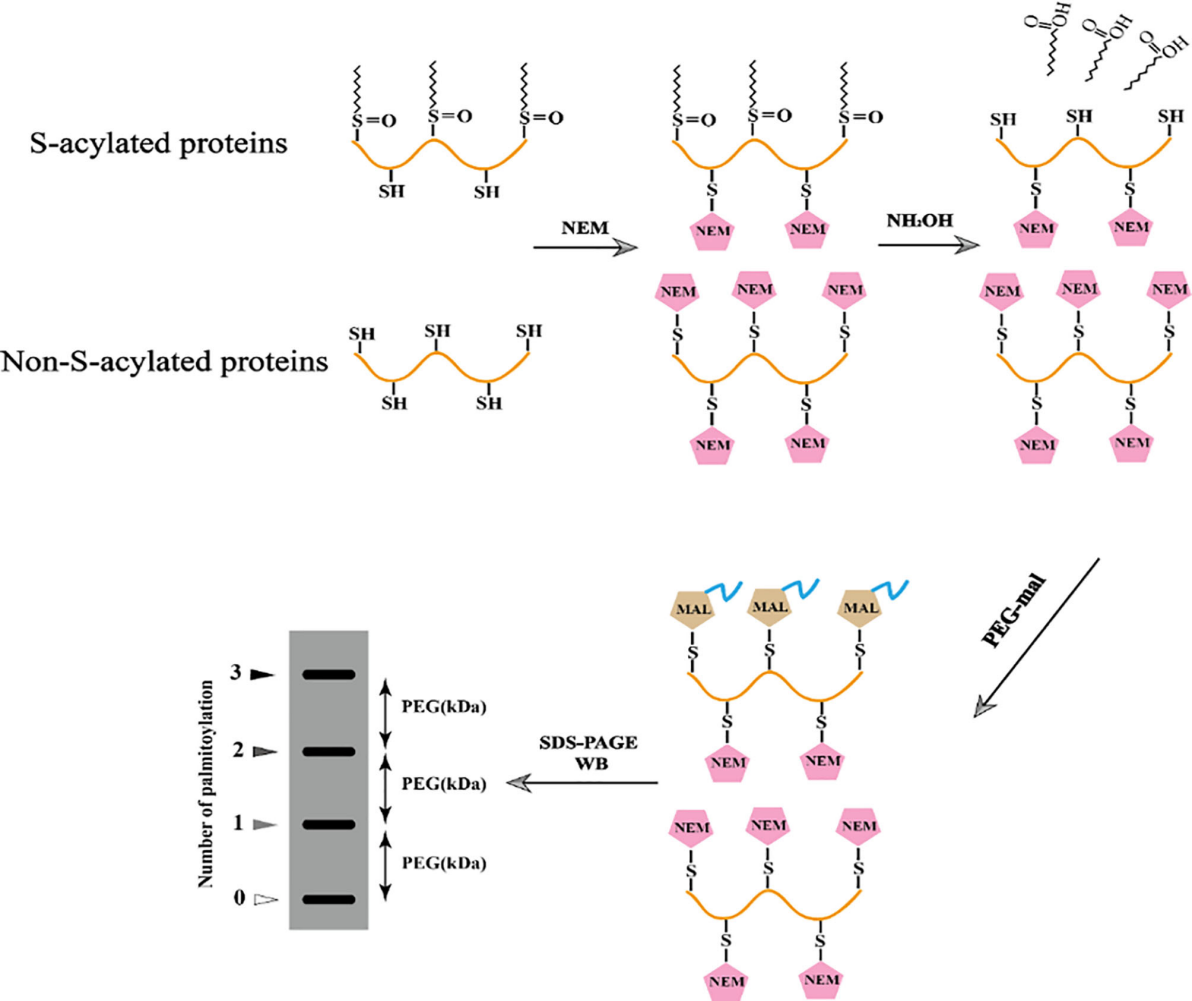
Schematic diagram of Acyl-PEGyl exchange gel-shift (APEGS) assay.

In 2016, a novel method based on Acyl-PEGyl exchange gel-shift (APEGS) was reported, in which the palmitoylated proteins were labeled with a fixed mass label, such as 2 kDa, 5 kDa, or 10 kDa methoxy polyethylene glycol maleimide (MPEG-mal) and detected by western blotting (Percher et al., 2016) (**Figure 2**).

**Figure 3:**
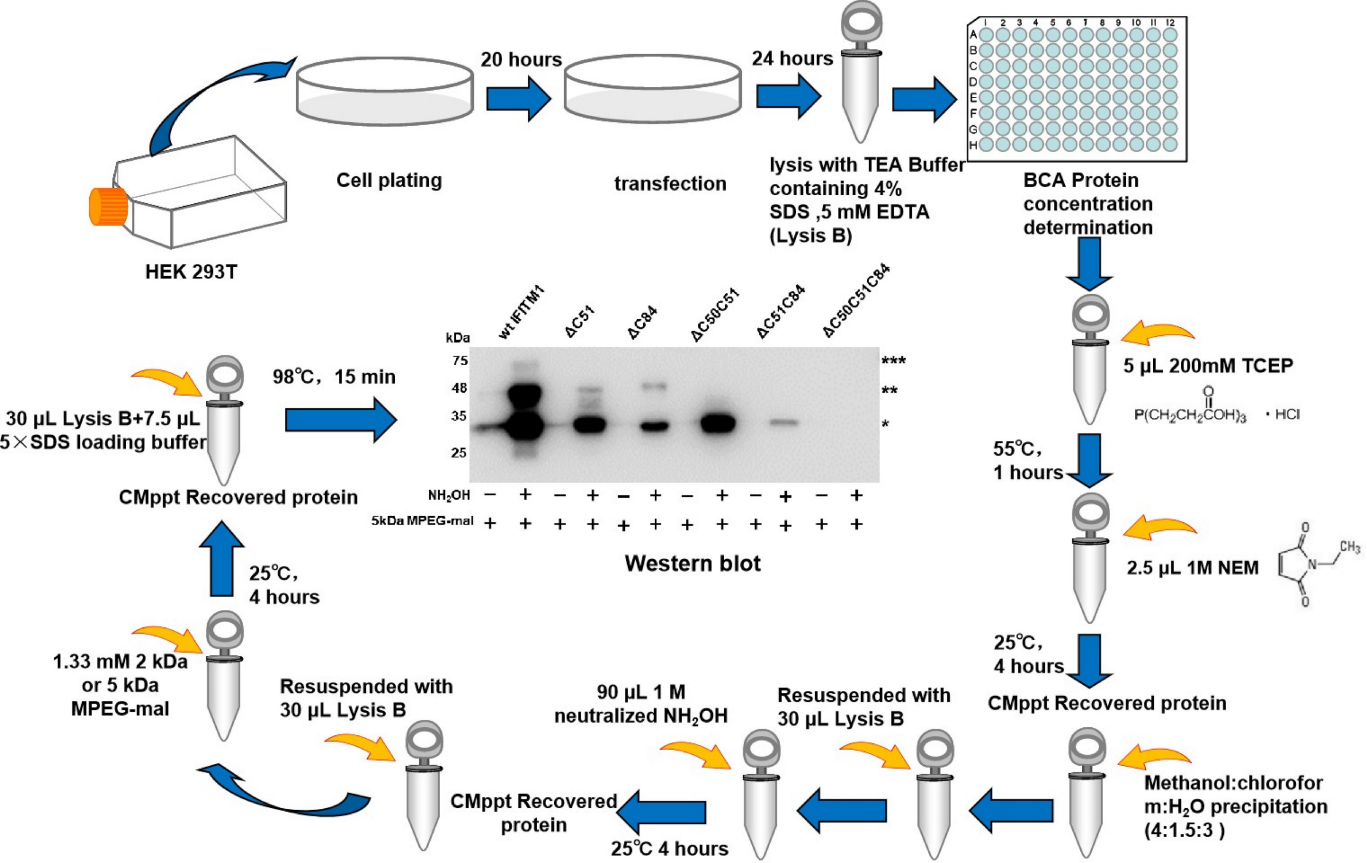
The diagram of the detailed procedures of APEGS for cell samples.

First, Tris (2-carboxyethyl) phosphine (TCEP) is used to reduce all disulfide bonds in cell lysate proteins. All non-palmitoylated cysteines or free hydroxyl groups in the protein sequence are blocked with N-ethylmaleimide (NEM). After activation with hydroxylamine hydrochloride (NH_2_OH), the cysteine sites where palmitoylation occurs are reduced to a free sulfhydryl state. Next, these free sulfhydryl groups are replaced by the newly added mass label, MPEG-mal, and finally, the palmitoylation is detected by western blotting. Due to its strong sensitivity and high specificity, this novel technology can determine the degree of palmitoylation and identify the number of palmitoylation sites of proteins with the help of gray-scale analysis software, such as the frequently used ones: Image J, Image-Pro Plus, and Quantity One. Based on the procedures of Takashi Kanadome (Kanadome et al., 2019) and Avital Perchera (Percher et al., 2016), we successfully identified three S-palmitoylation sites of swine IFITM1 and also analyzed the characteristics of each palmitoylation site by mathematical calculations of gray-scale analysis software (Xu et al., 2020) (**Figure 3**).

### 4.6 Other Supporting Strategies

Identification of palmitoylation sites by the above-mentioned methods, whether *in vivo* or *in vitro*, is usually time-consuming and labor-intensive. According to the characteristics of amino acids sequences and the structures of the palmitoylated proteins, researchers developed the software or websites to predict the possible palmitoylation sites for proteins. The in silico-based prediction platforms, such as CSS-Palm, IFS-Palm, WAP-Palm, PalmPred, SwissPalm database, and others, can narrow down the possible palmitoylation sites and can therefore guide further experimental designs. Here, we highlight two of them. The clustering and scoring strategy for palmitoylation sites prediction (CSS-Palm) system was implemented by Zhou et al., who developed a free accessible web server (Zhou et al., 2006; Ren et al., 2008). Since then, with CSS-Palm, many potential palmitoylated proteins of cells have been predicted, including PD-L1 (Zhou et al., 2019; Wang et al., 2020), MC1R (Chen et al., 2017), and differential S-acylation of enveloped viruses (Kordyukova et al., 2019). In 2021, a novel predictor, graphic presentation system for the prediction of S-palmitoylation (GPS-palm) was developed. This platform showed great improvement in the general prediction of S-palmitoylation sites compared with other existing tools (Ning et al., 2021). GPS-palm also provides two species-specific predictors for predicting human-and mouse-specific sites produced by the same research group would be useful tools for researchers of the related fields.

To confirm the amino acid sites of palmitoylation or to investigate the function or localization of palmitoylated proteins, mutagenesis is utilized as the standard and common approach. In this method, the potentially palmitoylated cysteine residue is replaced by serine or alanine. Because of its similar structure to cysteine, serine substitution can maintain the properties of putative palmitoylated protein; however, this substitution might cause side-chain defects and false positivity due to the higher hydrophilicity of hydroxyl groups.

## 5 Conclusion/Perspective

In the last few years, considerable headway has been made in the field of protein S-palmitoylation, including the increased number of the identified S-palmitoylated proteins, the drastically improved detecting methods, the developed labels, and some promising inhibitors. The scientific community has reached a consensus on the significance of palmitoylation in the occurrence and progression of certain diseases. Studies on protein palmitoylation have gained importance after the realization of the harmful effects caused by a variety of viruses. Quite a few researchers believe that palmitoylation-related proteins or enzymes are likely to be a breakthrough point in the treatment of major diseases in the future. Based on APEGS and pulse-chase protocol (click chemistry), a team of Harvard Medical School found that fatty acid and zDHHC19-mediated palmitoylation are key factors for STAT3 signal regulation (Niu et al., 2019), which provided important evidence of the relationship between palmitoylation and inflammation or cancer. The studies mentioned in this review indicate that palmitoylation affects the crucial function of proteins by regulating the interactions of proteins. Therefore, it is predicable that potential therapeutic targets for related diseases would be found with further studies on protein palmitoylation. Recently, an inhibitor of epithelial growth factor receptor (EGFR), DHHC20 antagonist, has been discovered and may develop into a drug candidate for treating patients with KRAS mutation tumors (Kharbanda et al., 2020).

Despite the recent progress in studies related to the palmitoylation of proteins associated with viral infections, there are many questions to be answered, for example, how the palmitoylation of virial proteins regulates viral replication dynamically. The following findings and speculations may provide important references for researchers in this field. The palmitoylation of influenza virus HA may serve as a raft-targeting signal, which can recruit HA to the plasma membrane and form a large raft structure (Leser and Lamb, 2005; Hess et al., 2007); thus, it provides a platform for the assembly and budding of the virions (Gerl et al., 2012). S-palmitoylation of influenza virus M2 may facilitate the shedding of virus particles from the plasma membrane and their budding (Schroeder et al., 2005). Efficient replication of CHIKV depends on the palmitoylation of two membrane-associated loops of nsP1. This is because the acylation leads to a hydrophobic state and helps in the electrostatic interaction of the protein with the inner leaflet of the plasma membrane; thus, enhancing viral replication (Zhang et al., 2021). A recent study reported that the palmitoylation of coronavirus proteins and the ensuing formation of complex lipid membranes were crucial to virus replication and assembly (Tanner and Alfieri, 2021), which undoubtedly provides a clue for digging into potential application of palmitoylated proteins, especially during the COVID-19 pandemic. Besides, we think that in-depth exploration of the following directions would contribute to understanding protein palmitoylation at a higher level:

- The exploration of the relationship between the intracellular molecular mechanisms or pathways of virus entry or replication and the palmitoylated proteins involved in order to discover the target whose palmitoylation/depalmitoylation cycle should be regulated.
- The identification of enzymes catalyzing the cycle of protein palmitoylation and screening of proteins that interact with palmitoylated proteins so that they could be utilized as targets to regulate the palmitoylation cycle. For example, till now, only zDHHC family of palmitoylases have been identified (Mitchell et al., 2006). Also, there are very few newly confirmed depalmitoylases, except for ABHD17 and ABHD10 (Lin and Conibear, 2015; Cao et al., 2019). APTs and PPTs that catalyze the cycle of protein palmitoylation in other mammalian species have not been reported yet.
- High-throughput methods for unbiased and efficient identification of palmitoylated proteins involved in the whole process of virus-host interactions should be improved. Due to the strong hydrophobicity of palmitoylated proteins, many challenges exist in the detection and analysis of such proteins. Moreover, this kind of modification usually occurs in low or medium abundance proteins, which makes the identification disjointed as no specific antibody can be used (Yang et al., 2010). All these bottlenecks suggest that more reliable and effective methods should be developed.
- The discovery of specific inhibitors of the enzymes participating in protein palmitoylation. Although 2-bromopalmitate had been recognized as an inhibitor of protein palmitoylation, it has widely been recognized to exert off-target effects by inhibiting other enzymes involved in lipid metabolism and is also cytotoxic to cells (Pedro et al., 2013). A new inhibitor of DHHC, cyano-myracrylamide (CMA), was reported recently, which has a broad-spectrum inhibitory effect on DHHC family members like 2-bromopalmitate but with less toxicity and off-target effects (Azizi et al., 2021).
- Analysis of the structures of enzymes and viral proteins involved in the palmitoylation/depalmitoylation cycle would be helpful for a better understanding of the subtle ways of host-virus interactions and designing new inhibitors or drugs.

This review gives a brief overview of protein palmitoylation and provides some theoretical foundation for further research on palmitoylation and the biological function of target proteins that are involved in various diseases, including viral infections. We hope more researchers will demonstrate their interesting findings based on varying functions of the palmitoylated proteins in the future.

## Author Contributions

JX designed research. JX and XL wrote the paper. XL and ZX draw the picture. LS contributed the table. LS, WL, and AL modified the paper. All authors contributed to the article and approved the submitted version.

## Funding

This project was financially supported by the National Natural Science Fund (U1804108), the Foundation of Henan Educational Committee (19A230005), and the Preferred Foundation for Returned Scholar from Overseas of Ministry of Human Resources and Social Security of China (Study abroad personnel and expert service center of Henan Province) (2017–02).

## Conflict of Interest

The authors declare that the research was conducted in the absence of any commercial or financial relationships that could be construed as a potential conflict of interest.

## Publisher’s Note

All claims expressed in this article are solely those of the authors and do not necessarily represent those of their affiliated organizations, or those of the publisher, the editors and the reviewers. Any product that may be evaluated in this article, or claim that may be made by its manufacturer, is not guaranteed or endorsed by the publisher.
